# Pure intervention effect or effect in routine health care - blinded or non-blinded randomized controlled trial

**DOI:** 10.1186/s12874-018-0549-z

**Published:** 2018-08-31

**Authors:** Antti Malmivaara

**Affiliations:** 1 Centre for Health and Social Economics, National Institute for Health and Welfare, Mannerheimintie 166, 00270 Helsinki, Finland; 2 Orton Orthopaedic Hospital and Orton Research Institute, Orton Foundation, Helsinki, Finland

**Keywords:** Randomized controlled trial, Blinding, Effectiveness, Cost-effectiveness, Arthroscopic partial meniscectomy, Clinical comparability, Applicability, Study question analysis

## Abstract

**Background:**

Randomized trials provide the most valid evidence of effectiveness of interventions. The study aims to determine the primary study question for randomized controlled trials; to evaluate the study questions in trials on effectiveness of arthroscopic meniscectomy for meniscal rupture of the knee; and to explore the clinical and research implications.

**Methods:**

Previous studies on benchmarking controlled trials were utilized. A literature search was undertaken to find the trials on arthroscopic surgery for meniscal rupture of the knee, data was extracted, and checked for accuracy twice.

**Results:**

The first question in RCTs is whether to assess the pure intervention effect, or intervention effect in routine health care circumstances. The former necessitates a double blinded design and the latter a non-blind design. The trials on arthroscopic meniscectomy of the knee showed considerable differences in study characteristics.

**Conclusions:**

The study question in RCTs on pure intervention effect dictates use of blinded design, while question of intervention effect in routine health care dictates use of non-blinded design. Blinding should not be considered a validity criterion when study question is on effectiveness in routine health care. When informing patients, the potential for other effects besides the pure intervention effect should be considered.

**Electronic supplementary material:**

The online version of this article (10.1186/s12874-018-0549-z) contains supplementary material, which is available to authorized users.

## Background

The experimental studies, particularly randomized controlled trials (RCTs) provide the least biased information on effectiveness of medical interventions and create the basis for systematic reviews on effectiveness of interventions [1]. Blinding of patients and care givers safeguards that knowledge of treatment allocation will not confound the effectiveness estimates [1]. However, the effect estimates may vary between blinded and non-blinded RCTs, and this variation may be dependent on the outcome. Perceived outcomes, for example pain, may be reduced by the placebo effect in non-blinded real-world circumstances, while some objective outcomes, e.g. mortality may show less difference between blinded vs. non-blinded comparisons [2, 3]. The effectiveness of arthroscopic partial meniscectomy (APM) for a ruptured meniscus of the knee has been under debate in scientific journals [4–10], and was thus chosen for obtaining empirical data for the current study.

The aims of this study were to find and operationalize the study question to be considered first in randomized controlled trials (RCTs); to assess consequences of this operationalization both conceptually and by using empirical data on effectiveness of arthroscopic meniscectomy for meniscal rupture of the knee; and to explore the consequent clinical and research implications.

## Methods

The present study utilized the methods for observational effectiveness studies, the benchmarking controlled trials (BCTs), where there is a need for a very detailed description of the study questions, selection of patients, characteristics of patients, interventions, and outcomes [11]. In BCTs the study question is always on effectiveness in routine health care. The BCT framework was used to assess which is the first study question to be asked in experimental studies, the randomized controlled trials (RCTs).

Literature search was undertaken to find all randomized controlled trials published in peer-reviewed journals assessing effectiveness of arthroscopic partial meniscectomy of the knee in comparison to any other non-pharmacological treatment, including sham surgery, among patients having knee pain, with at least one-year follow-up. Trials focusing on knee osteoarthrosis were excluded. The following key words were used: arthroscopic partial meniscectomy, randomized controlled trial, systematic review. Cochrane CENTRAL, Ovid MEDLINE, and Web of Science databases till October 2017 were used to find the eligible articles by the author, who checked the search findings to exclude misclassifications. The search strategy is described in Additional file 1.

The descriptive information in each trial concerning blinded vs non-blinded study design, selection of patients, and characteristics of patients, interventions and outcomes were extracted by the author, who rechecked twice the accuracy of the data. The study question characteristics were also depicted in a flow chart to show the similarities and differences between the studies.

## Results

**Fig. 1:**
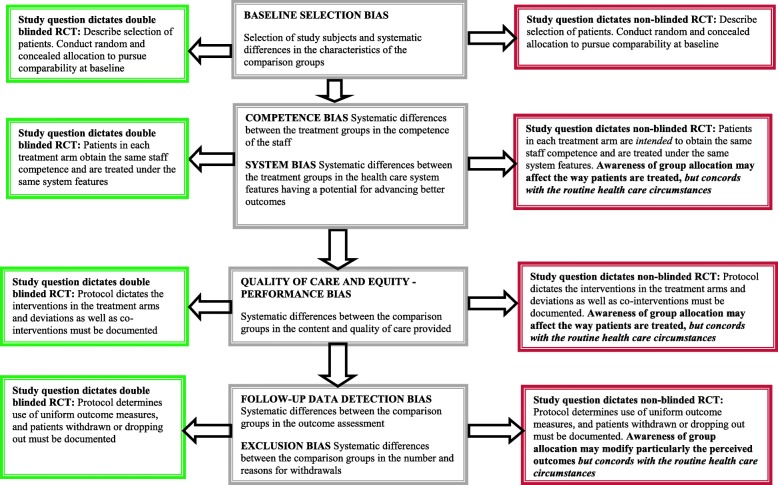
The validity issues in RCTs utilizing a double blinded design and in RCTs using a non-blinded design

**Fig. 2:**
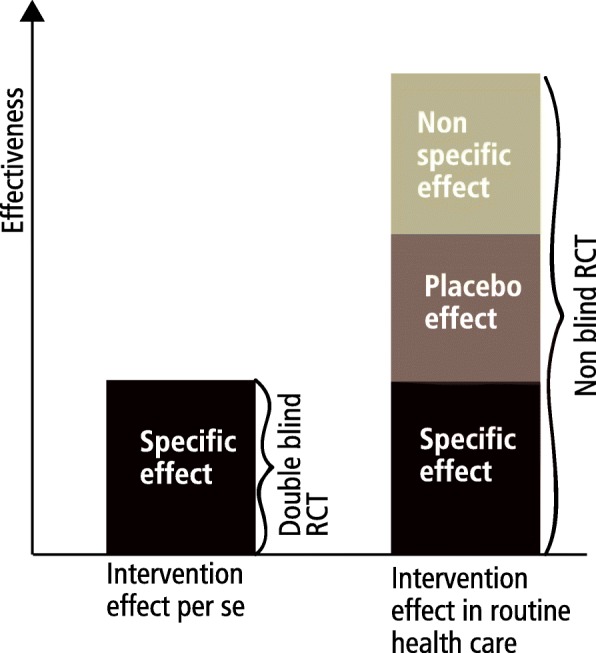
The components of the effect estimates of a double blinded design providing evidence of pure intervention effect and of a non-blinded design providing evidence of intervention effect in routine health care circumstances. The quantitative effects are illustrative and not based on empirical data

Characteristics of RCTs assessing pure intervention effect or effectiveness in real-world health care are shown in Table 1. The validity issues in RCTs utilizing a double blinded design and in RCTs using a non-blinded design are shown in Fig. 1. The double blinded design aims to assess the pure (specific) effects of an intervention, often biologic effects; while in routine health care the specific effect is complemented with a placebo effect and non-specific effects caused by the interaction between the patient and those providing care (Fig. 2).

**Table 1:** Characteristics of randomized controlled trials (RCTs) aiming to study effectiveness (or cost-effectiveness) of an intervention per se or effectiveness (or cost-effectiveness) of an intervention in routine health care circumstances

The first choice/appraisal to be made when planning/assessing a RCT → Characteristics of RCTs aiming to study effectiveness per se or in routine health care ↓	RCT aiming to study effectiveness of an intervention per se	RCT aiming to study effectiveness of an intervention in routine health care circumstances
Study design	Double-blinded study design	Non-blinded study design
Validity for assessing effectiveness of intervention per se	Valid design	Not a valid design
Validity for assessing intervention effectiveness in routine health care	Not a valid design	Valid design
Appropriateness for informing patients in routine health care; including use of number needed to treat (NNT) figures from double blinded RCTs	May give biased estimates, and in case of a placebo controlled trial, estimates may undervalue the effectiveness in routine health care	Gives non-biased estimates for intervention effectiveness in a particular routine health care; evidence is generalizable to similar patient, intervention, and health care contexts
Validity of cost-effectiveness estimates and incremental cost-effectiveness ratios (ICERs) in relation to routine health care	May give biased estimates, and in case of a placebo controlled trial, may undervalue the cost-effectiveness in routine health care	Gives non-biased estimates for intervention effectiveness in a particular routine health care.
Appropriateness of blinding of patients and health care providers as validity criteria of individual RCTs	Yes	No
Appropriateness for assessing efficacy (i.e. effectiveness in ideal circumstances) of interventions	Yes	Yes
Capability to provide effectiveness estimates applicable to everywhere anytime	No	No

### Study question dictates double blinded study design

When the study question is to assess the pure intervention effect the answer can be obtained only by a double blinded design (Table 1). The comparison can be between active intervention and placebo, or between two or more active interventions. When the comparison is to placebo, the study assesses the incremental effectiveness of the intervention beyond the effect caused by placebo. The study question in a double blinded randomized controlled trial is on pure (usually biological) effect of the treatment, while the clinical question in routine health care, where the patient and the care provider are always aware of the treatment options, is on what is the biological effect of the intervention plus the placebo effect of this intervention, Furthermore the overall effect is increased with non-specific treatment effects (information, advice, support) related to the intervention under study (Fig. 2).Therefore, double blind placebo controlled trials do not answer the question of effectiveness in routine health care. When two or more active interventions are compared with another in a double blind RCT the study assesses difference in effectiveness between the interventions, when placebo effect has been controlled by the double blinded design.

### Study question dictates non-blinded study design

In routine health care patients and their health care providers are always aware of the treatment options and to concord with this a non-blinded RCT is necessary (Table 1). When the comparison is between active intervention and no intervention (a choice to be made in clinical praxis), the study assesses the pure intervention effect plus its placebo effect plus non-specific treatment effects in comparison to none of these three effectiveness components (Fig. 2). Similarly, when the comparison is between two active interventions the study question again accords with the clinical question in routine health care, where in both groups the specific effect, placebo effect and non-specific effect add to the effectiveness estimates.

### The study questions in RCTs on arthroscopic partial meniscectomy

The primary literature search of the three databases found altogether 2375 abstracts of articles. Based on data in abstracts altogether 6 randomized controlled trials (RCTs) assessing effectiveness of arthroscopic partial meniscectomy and fulfilling the inclusion criteria where found (Table 2). [12–17]

**Table 2:** The main characteristics of the six randomised controlled trials assessing effectiveness of arthroscopic partial meniscectomy

Year, Study, country → ↓Study characteristics	2007 and 2013, Herrlin et al. [12] Sweden	2013, Katz et al. [13] USA	2013, Sihvonen et al. [14] Finland	2013, Yim et al. [15] South-Korea	2014, Gauffin et al. [16] Sweden	2016, Kise et al. [17] Norway
1. Study type						
1.1. Assessment of intervention effect per se	No	No	Yes	No	No	No
1.2. Assessment of real world effectiveness	Yes	Yes	No	Yes	Yes	Yes
2. Selection of patients						
2.1. Eligiblity criteria	45–64 years; knee pain without a trauma, daily or almost daily pain during last 2–6 months; knee osteoarthritis (Ahlbäck^c^ grade 0 or 1); medial meniscal tear on MRI	Symptomatic (at least four weeks) patients ≥45 years with a meniscal tear and osteoarthritis on MRI or radiography Excluded: Chronically locked knee, Kellgren-Lawrence grade 4	35 to 65 years; knee pain for > 3 months; clinical findings consistent with a tear of the medial meniscus and verified by MRI. Excluded: obvious traumatic onset or knee osteoarthritis (Kellgren-Lawrence^c^ grade > 1)	Degenerative horizontal tear of the posterior horn of the medial meniscus on MRI; daily knee pain Exclusion: a history of definite trauma	age 45–64, symptoms more than 3 months, Ahlbäck^c^ grade 0 in X-rays; had undergone prior physiotherapy	35–60 years; unilateral knee pain more than two months without a major trauma; medial degenerative meniscal tear by MRI; radiographic changes at most, grade 2 byKellgren-Lawrence^c^
2.2. Description of patients’ clinical path before being eligible	No	No^a^	No	No	Yes	No
2.3. Comprehensive population of catchment area	No	No	No	No	Yes	No
2.3. Place and time of recruitment. Number of patients per hospital year.	1 orthopedic clinic; June 2003 to Apr 2005 → 82 patients per hospital per year	7 tertiary centers; from June 2008 to Aug 2011 → 16 patients per hospital per year	5 orthopedic clinics; Dec 2007 to Jan 2013 → 6 patients per hospital per year	1 orthopedic clinic; Jan 2007 to July 2009. → 43 patients per hospital per year	1 orthopedic clinic; Mar 2010 to Apr 2012 → 71 patients per hospital per year	2 hospitals; Oct 2009 to Sep 2012 → 23 patients per hospital per year
2.4. Declining participation N (%)	40%	433/784 (55%)	24/205 (12%)	49/162 (30%)	5/179 (3%)	85/226 (38%)
2.5. Pre-intervention therapy	No	Medications, activity limitation or physical therapy for at least 4 weeks	Conventional conservative treatment	No	Physiotherapy (exercise) for at least 3 months	No
2.6. Verification of diagnosis	Clinically^b^ and by MRI	Clinically and by MRI	Clinically and by arthroscopy	Clinically and by MRI	Clinically	Clinically and by MRI
2.7. Osteoarthrosis (OA); based on X-rays/MRI	Yes or no OA^c^: X-rays only	Yes or no OA^c^: X-rays; Yes OA: MRI	No OA^c^: X-rays only	No OA^c^: X-rays only	No OA^c^: X-rays only	Yes or no OA^c^: X-rays only
2.8. History of a trauma as an exlusion or inclusion criterion	Excluded: Traumatic knee pain	Included: Sudden onset of symptoms; Excluded: locked knee	Excluded: Obvious traumatic onset	Excluded: History of definite trauma	Included: Sudden onset of symptoms; Excluded: locked knee	Excluded: Sudden onset of symptoms
3. Baseline characteristics (incl. Primary outcomes)						
3.1. Number of patients	180 (data on 90)	351	146	108 (data on 102)	150	140
3.2. Clinical important data^d^ Age, gender, outcomes: primary follow-up time; pain, disability, (*primary outcome*^j^: (APM/comparator)	56 yrs., 61% males; *KOOS pain* ^e^ *56/62 (median), VAS* ^f^ *at physical activity 6/5 (median); Lysholm* ^g^ *61/73*	59/58, 43% males; 6 months, *WOMAC*^h^ *(OA scale) 37/38,* KOOS pain 46/47	53 yrs., 61% males; *Lysholm*^i^ 60/60; *WOMET*^i^56/53; *Pain after exercise (NRS*^f^*) 5.8/6.1*	57 yrs., 21% males; *Pain VAS* ^f^ *5.2/4.9;* *Lysholm* ^i^ *64/65*	54 yrs., 73% males; *KOOS pain 55/58*	49/50 yrs., 61% males; 24 months; KOOS pain 68/63; *Knee function 64/58, KOOS*_*4*_ *score 54/60*
3.3. Health status/risk status	No	No	No	No	No	No
3.4. Comorbidity	No	No	No	No	No	No
3.5. Behaviour (e.g. lifestyle)	Yes (sports activity)	Yes (physical activity)	No	No	Yes (physical activity)	Yes (smoking)
3.6. Environment (e.g. work conditions)	Yes (physical activity at work)	No	No	No	No	No
3.7. Inequality (e.g. socioeconomic status)	No	No	No	No	No	Yes (education)
4. Interventions						
4.1. Content of the arthroscopic partial meniscectomy APM + possible co-interventions (N randomised)	APM 44, debridement 14, (90, data of 47) + Exercises twice a week, 8 weeks	APM, removing loose fragments of cartilage (174, data of 161)	APM (70)	APM 54 (3 patients with additional procedures^k^) + home exercise 8 weeks (54)	APM 56, other procedures 5, no procedures 8 + Unsupervised exercises (75)	APM (70)
4.2.Content of the comparison intervention (N)	Exercises twice a week, 8 weeks (90, data of 43)	Exercises 1–2 times weekly, 6 weeks (177, data of 169)	Sham APM (75)	Supervised exercises, thrice weekly for 3 weeks. Home exercise, 8 weeks (54)	Unsupervised exercises 2 times weekly 12 weeks (75)	Exercises 2–3 times weekly, 12 weeks (70)
4.3. Attendance in exercise therapy (%)	Unclear	91% attended 8 visits	Not applicable	Unclear	Unclear	43/70 (61%) at least satisfactory
4.4. Failure to obtain surgery (%)	0 (0%)	9/174 (5%) at one year	0 (0%)	1/54 (2%)	9 (12%) at one year	6/70 (9%)
4.5. Crossover to surgery (%)	13/46 (28%) during 24 months	59/177 (33%) at one year	Sham 5 (7%) (APM 2 (3%))	1/54 (2%) ^k^	16/75 (21%)	13/70 (19%)
4.6. Rehabilitation (additional)	Not reported	Not reported	Not reported	Not reported	Not reported	Not reported
4.7. Other use of health care services	Not reported	Not reported	Not reported	Not reported	Not reported	Not reported
4.8. Sickleaves	Not reported	Not reported	Not reported	Not reported	Not reported	Not reported
5. Outcomes						
5.1. Primary outcomes	6 months: KOOS^e^;Lysholm^g^; Tegner^l^; VAS^f^	12 months: WOMAC^h^	12 months: Lysholm^g^; WOMET^i^; Pain after exercise (NRS^f^)	24 months: VAS^f^; Lysholm^g^; Tegner^l^; patient satisfaction	12 months: KOOS^e^ pain scale	KOOS4 (4/5 of KOOS^e^ subscales); 3 months: thigh muscle strength
5.2. Follow-up percentage; reasons for dropping out reported: Yes/No	Exerc:96% (47/49) APM: 96% (45/47) No	Physiotherapy: 93% (164/177) APM: 90% (156/174) Yes	Sham surgery: 100% (76/76) APM:100% (70/70) No drop-outs	Non-operative: 96% (52/54) APM: 93% (50/54) Yes	Exerc:93% (70/75) APM: 80% (60/75) No	Exerc: 89% (62/70) APM: 91% (64/70) No
5.3. Outcomes assessed separately for disadvantaged patients (e.g. poor socioeconomic status)	No	No	No	No	No	No

*APM* arthroscopic partial meniscectomy, *OA* osteoarthrosis, *MRI* magnetic resonance imaging

^a^Selection to the tertiary centers not described

^b^History and physical examination

^c^Kellgren-Lawrence classification: 0–1 (Sihvonen, Yim), 0–2 (Kise) 0–3 (Katz); Ahlbäck classification: 0 (Gauffin); 0–1 (Herrlin)

^d^Mean values if not otherwise indicated

^e^KOOS (Knee injury and Osteoarthritis Outcome Score)

^f^VAS visual analog scale; NRS (numeral rating scale)

^*g*^Lysholm (Lysholm Knee Scoring Scale)

^*h*^WOMAC (Western Ontario and McMaster Universities Osteoarthritis Index

^*i*^WOMET (Western Ontario Meniscal Evaluation Tool)

^j^Primary outcome named by the authors

^k^The patient/s was/were withdrawn from the follow-up

^l^Tegner Activity scale; VAS/NRS: higher figures represent more severe pain or disability; Other outcomes: lower figures represent more severe pain or disability

All the 6 trials had reported appropriately the items needed for assessment of the study question. Information of patients’ selection process to the study was limited in five studies (Table 2). In one study, Gauffin et al., it was reported that more than 95% of patients were referred by the general practitioners to the study hospital from its catchment area19. The number of patients recruited per year per hospital varied from 6 to 82 between the studies. The proportion of eligible patients declining participation varied from 3 to 55%.

Very little or nothing was reported on general health status, comorbid conditions, behavioural factors like degree of physical activity, environmental factors like work conditions, or on degree of education or other socioeconomic factors of the patients (Table 2).

The trial by Gauffin et al. was the only study, which had a prerequisite of 12 weeks of exercises before eligibility of the patients was considered. In three studies previous exercise was not required before randomizing patients (Table 2).

In four studies crossover from conservative treatment to surgery varied from 19 to 36% during one year follow-up. In the studies by Sihvonen et al. and Yim et al. the cross-over was negligible.

Four studies used KOOS (Knee injury and Osteoarthritis Outcome Score) as their primary outcome; one study used WOMAC (Western Ontario and McMaster Universities Osteoarthritis Index), one study used visual analogue scale for pain and the Lysholm Knee Scoring Scale. Only one study used the meniscal lesion specific WOMET (Western Ontario Meniscal Evaluation Tool) as the primary outcome (Table 2). In one study no patient dropped out from the follow-up, in four trials at least 90% of patients did attend the primary follow-up; in one study (Gauffin et al.) 20% and 7% of patients were lost to follow-up in the exercise and APM groups, respectively.

**Fig. 3:**
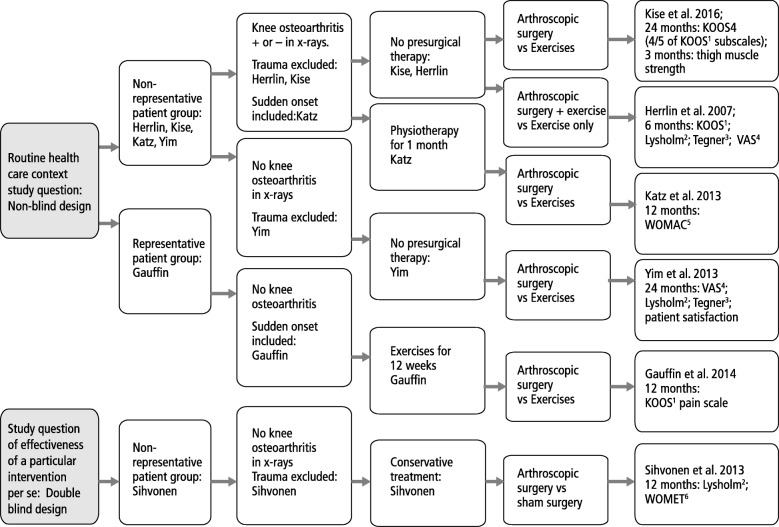
The study question analysis flowchart in randomized controlled trials on effectiveness of arthroscopic partial meniscectomy. The sequence is the following: Study population *→* Intervention effectiveness per se or intervention effectiveness in routine health care (blinded or non-blinded design) *→* Degree of selection (representative or non-representative study population) *→* Subcategory of the study population *→* Prior treatments before the experiment *→* Content of the index and reference interventions *→* Reference to the study; Primary outcome measures. ^1^ KOOS (Knee injury and Osteoarthritis Outcome Score); ^*2*^Lysholm (Lysholm Knee Scoring Scale): ^3^Tegner Activity Scale; ^4^VAS visual analog scale for pain; ^*5*^WOMAC (Western Ontario and McMaster Universities Osteoarthritis Index)*;*^*6*^WOMET (Western Ontario Meniscal Evaluation Tool)

Figure 3 shows in a flow chart the characteristics of the study questions: the pure intervention effect or effectiveness of intervention in routine praxis; representativeness of the study populations, the treatments before patients were considered eligible for the trial, the contents of the treatments in the actual experiment, and the primary outcome measures. Five of the RCTs had a non-blinded study design, and one had a double blinded design. Only one study, Gauffin et al., had recruited a comprehensive (and thus representative) patient population from its catchment area [16]. There were between study differences in the degree of concomitant osteoarthrosis of the knee. Gauffin et al. was the only study, which had 12 weeks of exercises tried before surgery. The content of the index and control interventions varied as well as the primary outcomes and their decisive time-points. In the trial by Gauffin et al., arthroscopic partial meniscectomy was more effective than exercise therapy. In the four other RCTs no treatment effectiveness was found. The only trial with a double blinded design, Sihvonen et al., found no effectiveness of arthroscopic partial meniscectomy in comparison with sham surgery.

## Discussion

The main finding of this paper is that *the study question* of assessing pure intervention effect *dictates* use of a double blinded design, and the study question of assessing effectiveness of intervention in routine health care settings *dictates* use of non-blinded RCT (Table 1, Fig. 1).

Table 2 and Fig. 3 show that all the RCTs on effectiveness of arthroscopic meniscectomy have studied a different study question, and therefore it was not possible to assess empirically the differences in effectiveness between those studies with non-blinded vs those with a blinded study design.

Double-blinded placebo controlled RCTs are needed to ensure that the intervention has favorable biological effects, and that the effects exceed the potential harms. If there is enough evidence that the intervention does not have any biological effect in a particular patient group, it may not be justified to study effectiveness of this intervention among similar patients in routine health care. Non-blinded RCTs are needed to gain evidence of effectiveness in routine health care circumstances, and double blinded RCTs, which have shown effectiveness of an intervention per se, should be followed by non-blinded RCTs to evaluate the effect in routine health care circumstances.

The effectiveness shown by a double blinded RCT is different from the effectiveness in ordinary health care circumstances, because in the latter placebo effects and non-specific treatment effects add to the pure intervention effect. Consequently, evidence of effectiveness from blinded placebo controlled RCTs, may not be as such valid for decisions in routine health care, and neither for informing patients. The blinded RCTs may underestimate effectiveness in routine health care, where placebo and non-specific effects add to the pure intervention effect; making also the number needed to treat figures biased. Therefore, when informing patients the potential for the placebo effect and non-specific effects to increase further the pure intervention effect should be taken into account.

These findings have implications also for health economics. The most valid way to obtain evidence on cost-effectiveness of a particular intervention is by an economic analysis alongside a randomized controlled trial [18]. However, if a double blinded design has been utilized, the effectiveness consists only of the effect of the pure intervention effect, and does not reflect the routine health care context, where there is also the placebo effect plus the effects provided by the interaction between the patient and the therapists. Thus, cost-effectiveness information from double blind RCTs with economic analysis should be followed by non-blinded trials to answer the routine health care study question. This applies also to modelling studies assessing the incremental cost-effectiveness ratios (ICERs): double blinded RCTs may not provide valid effectiveness estimates, as the placebo effect and other effects by routine health care are not considered. Thresholds for acceptable cost per one health related quality of life (HRQoL) are used in some countries like UK [19]. Again, the ICER estimates based on double blinded placebo-controlled trials may be biased and lead to larger costs per health-related quality of life (HRQoL), than actually occur in routine health care circumstances for which the estimates are intended.

Blinded RCTs are not the best way to assess efficacy (i.e. effectiveness in ideal circumstances) of an intervention, but ideal circumstances (meticulously selected patient population, most competent staff) can be designed equally well also for the non-blinded, routine health care experiments. Double blinded randomized trials are often conducted in optimal circumstances, and in these cases may reveal the best attainable effectiveness estimates, but they may also be conducted - as pragmatic trials - in routine health care contexts. Consequently, the idea of categorically denoting efficacy to double blind RCTs and effectiveness to non-blinded RCTs may not be justifiable.

The extent of the placebo effects and non-specific effects in routine health care may be dependent on the individual patient, and on the health care provider, and how well they are able to communicate between each other. Additional potential modifying factors may be e.g. competence of staff, structures of the health care system and cultural features [20]. There is a need to study these issues as modifiers of effectiveness in different patient groups and health care systems.

The assessment of risk of bias (internal validity) of the two study designs differs. When the study question is on intervention effectiveness per se, the success of double blinding and concealment of treatment allocation are of outmost importance. But, when the study aims to quantify the effectiveness of an intervention in routine health care circumstances, where both patients and health care staff are aware of the interventions chosen, *blinding is not justified*. Hitherto, the instructions for assessment of risk of bias in RCTs consider success of blinding in all RCT designs an important validity criterion [21]. The present paper argues that this interpretation is not tenable, and a distinction should be made between the two main study questions, which dictate whether to use blinded RCTs or non-blinded RCTs.

The study question analysis on arthroscopic meniscectomy of the knee shows major differences in the characteristics of the six trials, and all the trials are clinically heterogeneous. Therefore comparison of the double blinded vs non-blinded trials is not appropriate. The trial by Gauffin et al., which found effectiveness in routine health care, might be reproduced in a double blinded design with a sham surgery comparison to quantify the pure intervention effect in this representative patient population. In the four other non-blinded RCTs no treatment effectiveness was found, and considering the invasive nature of the intervention, with potential harms there may not be an indication to repeat these experiments in a double blinded design. In the only double blinded trial by Sihvonen et al. no benefits of surgery were found. As the surgery involves potential risks, there is no justification to proceed into non-blinded study design using same patient, intervention and outcome characteristics.

The study question analysis can be applied for planning and assessing of future RCTs, and for assessment of clinical homogeneity in systematic reviews.

## Conclusions

When the aim is to assess pure intervention effect, a double blinded RCT is indicated, and when the intention is to assess effectiveness of an intervention in routine health care circumstances, a non-blinded RCT is required. Appropriate blinding of patients and therapists is an essential validity criterion when assessing pure intervention effect, but blinding is contraindicated, when assessing effectiveness of interventions in routine health care. There is a need for non-blinded trials assessing effectiveness and cost-effectiveness in different patient groups and health care settings. When informing patients, the potential for additional effects besides the pure intervention effect should be considered. The study question analysis of the RCTs on arthroscopic meniscectomy of the knee showed that all the trials are clinically heterogeneous, and do not allow a meta-analysis or a comparison of the double blinded vs non-blinded trials.

## Additional file

Additional file 1: Literature search strategy. (DOC 40 kb)

## Abbreviations

BCT: Benchmarking Controlled Trial
HRQoL: Health Related Quality of Life
ICER: Incremental cost-effectiveness ratio
RCT: Randomized Controlled Trial
