# Supplementary material for: Pure intervention effect or effect in routine health care - blinded or non-blinded randomized controlled trial
Source: BMC Med Res Methodol. 2018 Aug 31;18:91. doi: 10.1186/s12874-018-0549-z (PMC6119259; doi:10.1186/s12874-018-0549-z)
Supplement: Supplementary file 1 — Literature search strategy. (DOC 40 kb) [file 12874_2018_549_MOESM1_ESM.doc]

# Appendix 1. Literature search strategy

# Literature search

## Searched databases

CENTRAL – Cochrane Library/Wiley Online Library
Web of Science – Core collection (Indexes=SCI-EXPANDED)
Ovid MEDLINE(R) Epub Ahead of Print, In-Process & Other Non-Indexed Citations, Ovid MEDLINE(R) Daily and Ovid MEDLINE(R) <1946 to Present>

## Search strategies

### CENTRAL - Cochrane Library/Wiley Online Library

Search Name: meniscus-surgery
Date Run: 12/10/17 12:27:36.805
Description:

ID Search Hits
#1 MeSH descriptor: [Meniscus] explode all trees and with qualifier(s): [Surgery - SU] 145
#2 MeSH descriptor: [Arthroscopy] explode all trees 1454
#3 MeSH descriptor: [Knee] explode all trees 706
#4 #2 and #3 112
#5 meniscal tear:ti,ab,kw (Word variations have been searched) 138
#6 arthroscopic meniscectomy:ti,ab,kw (Word variations have been searched) 167
#7 arthroscopic debridement:ti,ab,kw (Word variations have been searched) 104
#8 #1 or #4 or #5 or #6 or #7 in Trials 498

### Web of Science – Core Collection

#1 TS=(menisc*AND (surgery OR surgical* OR operati* OR arthroscop* OR injury OR injuries OR tear* OR torn)) OR TS=(knee* NEAR/3 arthroscop*) OR TS=("Arthroscopic debridement*") OR TS=(arthroscop* NEAR/3 meniscectom*)
#2 TS=(random* OR rct OR cct OR "controlled trial*" OR "control group*" OR double-blind* OR "double blind*" OR single-blind* OR "single blind*" OR cross-over OR crossover OR "cross over" OR placebo)
#2 AND #1
Results: 1,649
#2 AND #1
Refined by: DOCUMENT TYPES: ( ARTICLE OR CORRECTION OR REVIEW OR RETRACTED PUBLICATION OR PROCEEDINGS PAPER )
Results: 1,606
Indexes=SCI-EXPANDED Timespan=All years
(from Web of Science Core Collection)

### Database: Ovid MEDLINE(R) Epub Ahead of Print, In-Process & Other Non-Indexed Citations, Ovid MEDLINE(R) Daily and Ovid MEDLINE(R) <1946 to Present>

Search Strategy: 20/10/2017

--------------------------------------------------------------------------------

1 exp Meniscus/su [Surgery] (3976)
2 exp Arthroscopy/ (22577)
3 exp Knee/ (13573)
4 2 and 3 (450)
5 (menisc* adj5 (surgery or surgical* or operati* or arthroscop* or injury or injuries or tear* or torn)).ti,ab. (7325)
6 (knee* adj3 arthroscop*).ti,ab. (3229)
7 Arthroscopic debridement*.ti,ab. (840)
8 (arthroscop* adj3 meniscectom*).ti,ab. (780)
9 1 or 4 or 5 or 6 or 7 or 8 (12430)
10 Randomized Controlled Trials as Topic/ (121907)
11 randomized controlled trial/ (497319)
12 Random Allocation/ (99691)
13 Double Blind Method/ (157599)
14 Single Blind Method/ (26594)
15 clinical trial/ (548114)
16 clinical trial, phase i.pt. (20030)
17 clinical trial, phase ii.pt. (32310)
18 clinical trial, phase iii.pt. (15306)
19 clinical trial, phase iv.pt. (1629)
20 controlled clinical trial.pt. (99268)
21 randomized controlled trial.pt. (497319)
22 multicenter study.pt. (249307)
23 clinical trial.pt. (548114)
24 exp Clinical Trials as topic/ (332342)
25 or/10-24 (1316051)
26 (clinical adj trial$).tw. (321698)
27 ((singl$ or doubl$ or treb$ or tripl$) adj (blind$3 or mask$3)).tw. (167825)
28 PLACEBOS/ (36433)
29 placebo$.tw. (209223)
30 randomly allocated.tw. (25083)
31 (allocated adj2 random$).tw. (28243)
32 or/26-31 (583883)
33 25 or 32 (1542791)
34 case report.tw. (275614)
35 letter/ (1023624)
36 historical article/ (356118)
37 or/34-36 (1640796)
38 33 not 37 (1507650)
39 9 and 38 (1437)

## The Number of References by Database

| Dtabase | References |
| --- | --- |
| CENTRAL  *October 13^th^, 2017* | 498 |
| Web of Science *October 17^th^, 2017* | 1,606 |
| Ovid MEDLINE(R) Epub Ahead of Print, In-Process & Other Non-Indexed Citations, Ovid MEDLINE(R) Daily and Ovid MEDLINE(R) <1946 to Present> *October 20^th^, 2017* | 1 437 |
| Total | 3541 |
| References, duplicates excluded | 2375 |
